# A Prediction Equation to Estimate Vascular Endothelial Function in Different Body Mass Index Populations

**DOI:** 10.3389/fcvm.2022.766565

**Published:** 2022-03-10

**Authors:** Xiao Li, Hanying Liu, Yan Zhang, Yanting Gu, Lianjie Sun, Haoyong Yu, Wenkun Bai

**Affiliations:** ^1^Department of Ultrasound in Medicine, Shanghai Jiao Tong University Affiliated 6th People's Hospital, Shanghai Institute of Ultrasound in Medicine, Shanghai, China; ^2^Department of Endocrinology and Metabolism, Shanghai Jiao Tong University Affiliated Sixth People's Hospital, Institute of Shanghai Diabetes, Shanghai, China; ^3^Department of Cardiovascular Surgery, The Affiliated Hospital of Qingdao University, Qingdao, China; ^4^Department of Ultrasound in Medicine, South Hospital of Shanghai Jiao Tong University Affiliated 6th People's Hospital, Shanghai, China

**Keywords:** flow-mediated dilation, prediction equation, vascular endothelial function, atherosclerosis, body mass index

## Abstract

**Objective:**

Vascular endothelial dysfunction is considered an early predictor of endothelial injury and the initiating factor of atherosclerosis (AS). Brachial artery flow-mediated dilation (FMD) can detect endothelial injury early and provide important prognostic information beyond traditional cardiovascular (CV) risk factors. This study aimed to find the influencing factors of FMD and develop a simple prediction model in populations with different body mass indices (BMIs).

**Methods:**

In total, 420 volunteers with different BMIs were recruited in our study. Subjects were randomly assigned to the derivation and validation cohorts (the ratio of the two was 1:2) with simple random sampling. The former was used for influencing factors searching and model construction of FMD and the latter was used for verification and performance evaluation.

**Results:**

The population was divided into two groups, i.e., 140 people in the derivation group and 280 people in the verification group. Analyzing in the training data, we found that females had higher FMD than males (*p* < 0.05), and FMD decreased with age (*p* < 0.05). In people with diabetes, hypertension or obesity, FMD was lower than that in normal individuals (*p* < 0.05). Through correlation analysis and linear regression, we found the main influencing factors of FMD: BMI, age, waist-to-hip radio (WHR), aspartate aminotransferase (AST) and low-density lipoprotein (LDL). And we developed a simple FMD prediction model: FMD = −0.096BMI−0.069age−4.551WHR−0.015AST−0.242LDL+17.938, where R^2^ = 0.599, and adjusted R^2^ = 0.583. There was no statistically significant difference between the actual FMD and the predicted FMD in the verification group (*p* > 0.05). The intra-class correlation coefficient (ICC) was 0.77. In a Bland-Altman plot, the actual FMD and the predicted FMD also showed good agreement. This prediction model had good hints in CV risk stratification (area under curve [AUC]: 0.780, 95 % confidence intervals [95% CI]: 0.708–0.852, *p* < 0.001), with a sensitivity and specificity of 73.8 and 72.1%, respectively.

**Conclusions:**

Males, older, obesity, hypertension, diabetes, smoking, etc. were risk factors for FMD, which was closely related to CV disease (CVD). We developed a simple equation to predict FMD, which showed good agreement between the training and validation groups. And it would greatly simplify clinical work and may help physicians follow up the condition and monitor therapeutic effect. But further validation and modification bears great significance.

## Introduction

AS is the most common pathological mechanism of coronary artery disease, peripheral artery disease and cerebrovascular disease (1). The chronic accumulation of subendothelial atherosclerotic plaque or the shedding of unstable plaque will cause luminal stenosis to restrict blood flow and cause severe tissue hypoxia, eventually causing myocardial infarction and stroke, which is the most common cause of death in the world (2). Vascular endothelial dysfunction is generally considered an early predictor of endothelial injury and the initiating factor of AS (3). Thus, early recognition of endothelial injury can help take intervention measures as soon as possible to effectively delay or even reverse the process of AS.

The evaluation methods of endothelial function are mainly divided into invasion and non-invasion. In the past, invasive inspections such as coronary angiography and forearm plethysmography (4) were often used. However, these invasive techniques were burdensome for the subjects with low repeatability and high risk characteristics. Especially for healthy people or asymptomatic patients, it was usually inappropriate to perform these examinations. Therefore, some non-invasive technologies have emerged with higher repeatability and accurate and reliable results. Carotid intima media thickness (CIMT) measurement is an effective method for the early assessment of AS (5). With its simple operation and low price, the ultrasound measurement of CIMT is widely used in the clinical screening of vascular diseases. However, there is interobserver variability in measurement, and its accuracy must be improved. Peripheral arterial tonometry (PAT) is an effective method to evaluate the microvascular function (6). The vasomotor function is evaluated by vasodilation after compression, and the results obtained by its automatic detection program are more reliable. Correspondingly, FMD is currently the most commonly used method to evaluate the endothelial function (7). Its result is accurate, reliable and highly repeatable, and it is considered the most important method to non-invasively evaluate the endothelial function.

However, there are many problems in the application of FMD in daily clinical practice. With its high requirements for operation skills, it requires well-trained operators. The discomfort caused by prolonged pressurization makes it unacceptable. The inspect equipment is also expensive, especially in primary hospitals, which results in much more difficult assessments.

Thus, we developed a prediction model based on FMD results with anthropometric measurements and laboratory analysis to facilitate the evaluation of endothelial damage in populations with different BMIs. It was helpful to popularize at primary hospitals and convenient for early detection and early intervention, which we hope to improve the prognosis of atherosclerosis.

## Methods

### Participant Selection and Design

This study consequently enrolled subjects from Jan 2021 to April 2021 in the Sixth People's Hospital Affiliated to Shanghai Jiao Tong University, China. Patients with the following conditions were excluded from our study: current malignancy with a life expectancy <6 months; undergoing radiotherapy, chemotherapy, or molecular targeted therapy; severe mental illness or language barriers that prevented normal communication with researchers. In total, 420 subjects aged 18–60 years were recruited, including 108 males and 312 females, all of whom were Chinese. FMD and anthropometric measurements and laboratory analysis were performed on all participants. Hypertension was defined as > 140/90 mm Hg according to the 2018 European Society of Cardiology (ESC)/European Society of Hypertension (ESH) guidelines (8). Criteria for the diagnosis of diabetes was fasting blood glucose (FBG) ≥ 7.0 mmol/L or 2-h plasma glucose value ≥ 11.1 mmol/L during a 75-g oral glucose tolerance test or the glycated hemoglobin (HbA1c) ≥ 6.5% or patient with classic symptoms of hyperglycemia or hyperglycemic crisis, a random plasma glucose ≥ 11.1 mmol/L (9). Given that total cholesterol (TC) and/or high-density lipoprotein (HDL) are included as risk assessment factors in many guidelines on the assessment of cardiovascular risk (10–12), we tentatively classified TC ≥ 5.2 mmol/l or HDL < 1.0 mmol/l as the dyslipidemia group for ease of analysis. Then, the population was randomly assigned to the derivation and validation cohorts (the ratio of the two was 1:2) with simple random sampling to search for the influencing factors of FMD and construct and verify the equation. The protocol was approved by the Shanghai Jiao Tong University Ethics Committee and conformed to the Declaration of Helsinki. All subjects provided written informed consent to participate in the study.

### FMD and CIMT Measurement

FMD was measured according to the guideline (13) with an Omron UNEX EF 38G (UNEX Corporation, Nagoya, Japan) tester, and the probe frequency was 10 MHz. Prior to the FMD examination, subjects should fast (>6 h), avoid exercise (>24 h), and refrain from caffeine, vitamin C and alcohol (>12 h). Smokers must refrain from smoking for >6 h. When subjects take drugs, they should wait 4 times the half-life of the drug. Then, they should be studied in a quiet, temperature-controlled room and relaxed for at least 10 min to relieve mental stress and physical activity. The blood pressure was measured twice on the right arm in the sitting position, and the average value was obtained. Then, in the supine position with the right arm extended, the cuff was placed on the forearm, and the brachial artery was scanned 3–5 cm above the antecubital fossa. We used an H-shaped probe capturing two short-axis and one long-axis for automatic probe position correction to identify clear vascular boundaries. And a probe-holding device was used to further ensure optimal image. First, the baseline inner diameter of brachial artery was measured; then, the cuff was inflated to a pressure of 50 mmHg higher than the subject's systolic blood pressure and held for 5 min. Afterwards, the cuff was deflated, and the diameter of the artery was measured again over the next 3 min to capture the peak brachial artery diameter. The continuous measurement process was monitoring by automatic edge-detections systems. Finally, the FMD result was automatically calculated by the machine: FMD = [(maximum diameter – diameter at rest)/diameter at rest] × 100 (%). FMD was measured twice for each participant at an interval of 20 min, and the average value was obtained.

CIMT was performed using a MINDRAY DC-8 or a MINDRAY DC-80S ultrasound machine with a 10-MHz linear probe. Patients were examined in the supine position with a slight rotation of the neck to the contralateral side. CIMT was defined as the distance between the lumen-intima boundary and the media-adventitia boundary. And it was measured at ~1 cm posterior to the common carotid artery bifurcation at a site with uniform intima without plaque lesions. The proximal and distal walls were measured and averaged. Finally, the average value of the left and right common carotid arteries was taken as the average CIMT.

The entire inspection project for FMD and CIMT was completed by two experienced physician with at least 3 years of operating experience and the physicians did not know any information about them.

### Clinical and Laboratory Analysis

Operators used a digital scale to measure the height and weight of participants, who had to take off shoes and wear light clothes. Then, we calculated the BMI = body weight (kg)/height squared (m^2^). The waist circumference was measured using tape around the abdomen through the midpoint of the lower edge of the costal arch and the anterior superior spine by a trained examiner. Then, the subjects stood upright with legs close together, and the tape was placed horizontally on their front pubic symphysis and the most convex part of the back gluteus maximus to measure the hip circumference. And the WHR was calculated using the formula: WHR = waist circumference (cm)/hip circumference (cm). The systolic blood pressure (SBP) and diastolic blood pressure (DBP) were measured twice in a quiet state for 1 day, and mean values were used for analysis. Mean arterial pressure (MAP) was expressed as MAP = (SBP+2 × DBP)/3.

We searched for cardiovascular risk factors proposed by some guidelines, and on this basis, added variables that may be associated with FMD for model construction: triglyceride (TG), TCHDL, LDL, alanine aminotransferase (ALT), AST, γ-glutamyl transpeptidase (γ-GT), alkaline phosphatase (ALP), free triiodothyronine (FT_3_), free tetraiodothyronine (FT_4_), thyroid stimulating hormone (TSH), prealbumin (PAB), total bile acid (TPA), total bilirubin (TBiL), direct bilirubin (DBiL), serum creatinine (Scr), serum uric acid (SUA), blood urea nitrogen (BUN), retinol-binding protein (RBP), cystatin C (CysC), FBG, HbA1c, insulin, and C-peptide (CP). Venous blood samples were drawn from all subjects in the early morning after 8 h of fasting. All biochemical determinations were performed in the same laboratory using standard laboratory methods. Insulin was measured by radioimmunoassay (ADVIA Insulin Ready Pack 100, Bayer Diagnostics, Milan, Italy) with intra- and inter-assay coefficients of variation < 5%.

### Statistical Analysis

Statistical analyses were performed with the SPSS software, version 26 and MedCalc software, 20.0.3. The normality of distribution was checked with Kolmogorov–Smirnov test. Normally distributed data are expressed as the mean ± standard deviation, whereas data not normally distributed are expressed as the median and interquartile range. Categorical variables were expressed as percentages. Independent two-sample *t*-test and non-parametric test (Mann–Whitney) were used for comparison of quantitative data. Pearson's chi-square (χ2) was used to compare categorical data. We first analyzed the difference of FMD among subgroups of different genders, different ages and with or without hypertension, diabetes or dyslipidemia. And then we further searched for the influencing factors of FMD through correlation analysis (*p* < 0.01, *r* > 0.3) and linear regression (*p* < 0.01), and constructed a FMD prediction model based on multivariate linear regression (stepwise). Finally, validation and performance evaluation were performed in the validation sample by ICC and Bland–Altman plot. In addition, we analyzed the value of our predictive equation in identifying risk factors for CVD through receiver operating characteristic (ROC) curves. A *p* < 0.05 indicated statistical significance.

## Results

### Baseline Characteristics and Within-Group Analysis

In total, 420 subjects fulfilled the inclusion criteria and were divided into a derivation group and a verification group by random sampling. The ratio of the two was 1:2, i.e., 140 people in the derivation group and 280 people in the validation group. The demographic and clinical characteristics of the two groups are shown in Table 1. There was no statistically significant difference between them (*p* > 0.05).

**Table 1:** Characteristics in the derivation and validation cohorts.

**Characteristics**	**Derivation**	**Validation**	***P*-value**
	**data set**	**data set**	
N (M/F)	140 (38/102)	280 (70/210)	0.64
Age (years)	32.1 ± 8.1	31.3 ± 7.4	0.34
BMI (kg/m^2^)	35.5 ± 8.3	35.3 ± 8.7	0.81
HR (beats/min)	86.9 ± 12.7	86.5 ± 12.6	0.75
SBP (mmHg)	130.6 ± 19.7	128.7 ± 17.3	0.32
DBP (mmHg)	85.8 ± 12.9	84.8 ± 12.3	0.42
MAP (mmHg)	100.7 ± 14.2	99.1 ± 14.3	0.26
WHR	1.0 (1.0, 1.0)	1.0 (0.9, 1.0)	0.78
CIMT (mm)	0.6 (0.5, 0.7)	0.6 (0.5, 0.7)	0.23
TG (mmol/l)	1.5 (1.1, 2.4)	1.5 (1.1, 2.3)	0.68
TC (mmol/l)	5.2 (4.5, 6.1)	5.2 (4.4, 5.9)	0.71
HDL cholesterol (mmol/l)	1.2 (1.1, 1.4)	1.3(1.1, 1.4)	0.59
LDL cholesterol (mmol/l)	3.2 ± 0.9	3.2 ± 0.8	0.71
ALT (U/L)	43.0 (27.5, 77.5)	42.5 (25.0, 74.2)	0.93
AST (U/L)	25.0 (18.0, 44.0)	25.5 (18.0, 41.5)	0.91
γ-GT (U/L)	41.0 (27.0, 59.5)	36.0 (23.0, 56.3)	0.37
ALP (U/L)	76.0 (62.5, 92.5)	76.0 (64.0, 93.3)	0.62
FT_3_ (pmol/L)	5.1 (4.7, 5.4)	5.0 (4.6, 5.5)	0.55
FT_4_ (pmol/L)	16.3 (15.1, 17.4)	15.9 (14.8, 17.5)	0.76
TSH (mIU/L)	2.2 (1.5, 3.1)	2.5 (1.6, 3.6)	0.05
PAB (mg/L)	273.0 (241.0, 379.0)	272.5 (231.8, 304.0)	0.19
TBA (μmol/L)	2.9 (2.0, 5.4)	3.3 (2.1, 5.3)	0.30
TBiL (μmol/L)	8.7 (6.4, 11.5)	8.5 (6.3, 11.2)	0.28
DBiL (μmol/L)	2.2 (1.6, 3.0)	2.1 (1.7, 2.8)	0.61
Scr (mg/dL)	60.0 (52.1, 71.9)	60.6 (54.0, 68.9)	0.95
SUA (mg/dL)	402.0 (330.5, 453.5)	417.5 (346.8, 464.8)	0.27
BUN (mmol/L)	4.6 (3.9, 5.4)	4.6 (3.9, 5.4)	0.89
RBP (mg/L)	38.0 (32.0, 45.0)	36 (31.0, 42.0)	0.15
CysC (mg/L)	0.7 (0.6, 0.8)	0.7(0.6, 0.8)	0.84
FBG (mmol/l)	5.7 (5.0, 7.2)	5.6(5.1, 6.9)	0.63
HbA1c (%)	5.8 (5.4, 6.8)	5.8 (5.4, 6.7)	0.71
Insulin (μIU/ml)	25.9 (17.6, 38.4)	28.8 (19.9, 44.3)	0.31
CP (ng/ml)	3.9 (3.2, 4.8)	4.1 (3.4, 5.1)	0.56
Current smokers (%)	32 (22.9%)	48 (17.1%)	0.18
Hypertension (%)	30 (21.4%)	38 (13.6%)	0.06
Participants on	18 (12.9%)	29 (10.4%)	0.44
anti-hypertensives (%)			
Diabetes (%)	40 (28.6)	75 (26.8%)	0.70
Participants on	17 (12.1%)	39 (13.9%)	0.61
anti-diabetics (%)			
Dyslipidemia (%)	78 (55.7%)	152 (54.3%)	0.78
Participants on	2 (1.4%)	1 (0.4%)	0.22
lipid-lowering drugs (%)			
FMD (%)	6.4 (5.6, 7.6)	6.2 (5.4, 7.0)	0.51

*Data are shown as the mean ± SD or the median (interquartile range)*.

*M/F, male/female; BMI, body mass index; HR, heart rate; SBP, systolic blood pressure; DBP, diastolic blood pressure; MAP, mean arterial pressure; WHR, waist-to-hip ratio; CIMT, carotid intima media thickness; TG, triglyceride; TC, total cholesterol; HDL, high-density lipoprotein; LDL, low-density lipoprotein; ALT, alanine aminotransferase; AST, aspartate aminotransferase; γ-GT, γ-glutamyl transpeptidase; ALP, alkaline phosphatase; FT_3_, free triiodothyronine; FT_4_, free tetraiodothyronine; TSH, thyroid stimulating hormone; PAB, prealbumin; TBA, total bile acid; TBiL, total bilirubin; DBiL, direct Bilirubin; Scr, serum creatinine; SUA, serum uric acid; BUN, blood urea nitrogen; RBP, retinol-binding protein; CysC, cystatin C; FBG, fasting blood glucose; HbA1c, glycated hemoglobin; CP, C-peptide; FMD, flow-mediated dilation*.

**Figure 1:**
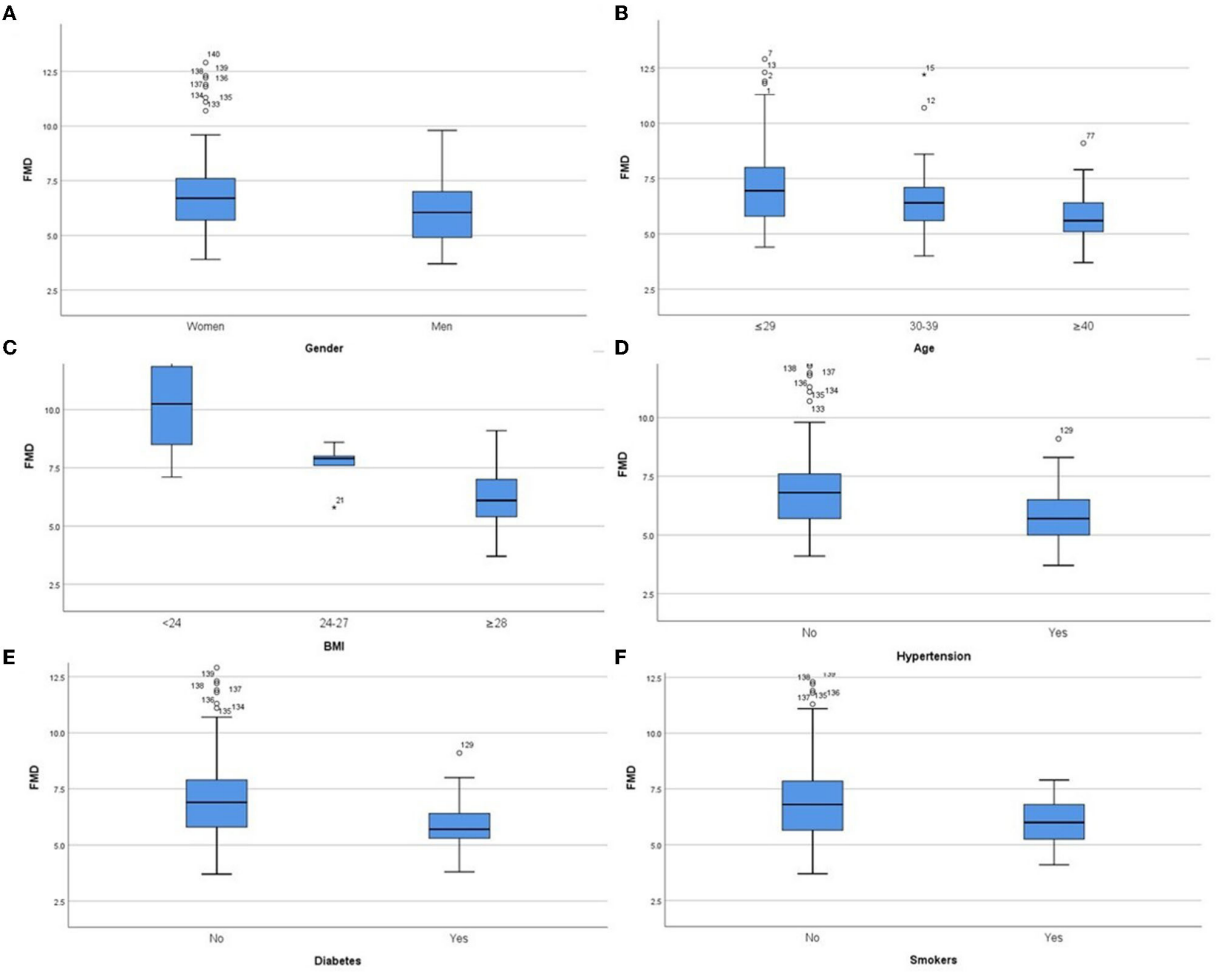
Comparison of FMD values in subgroups of both genders **(A)**, different ages **(B)**, different BMIs **(C)**, hypertension **(D)**, diabetes **(E)** and smoking **(F)**. *P* < 0.05 in all subgroups.

**Figure 2:**
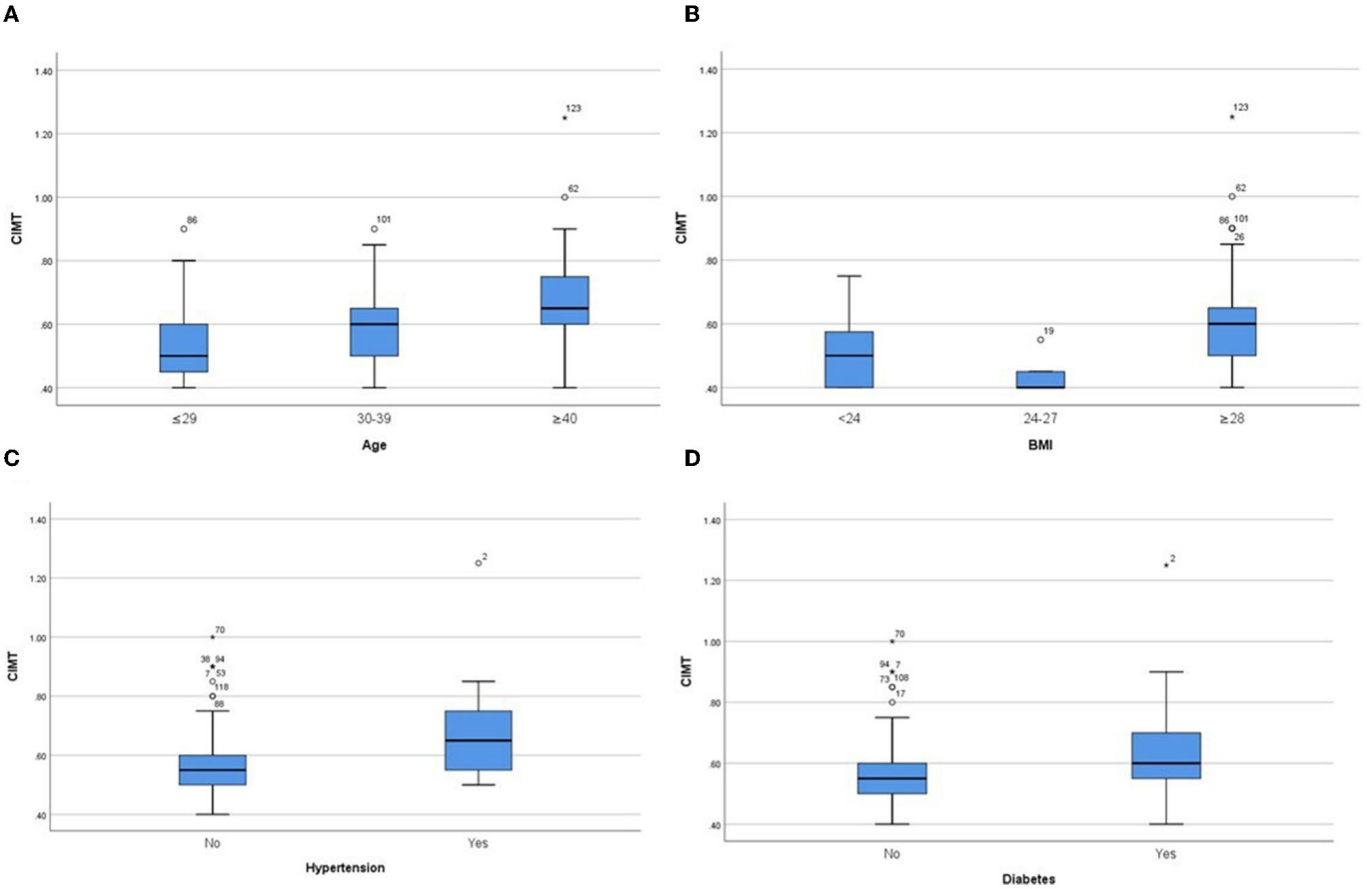
Comparison of CIMT values in subgroups of different ages **(A)**, different BMIs **(B)**, hypertension **(C)** and diabetes **(D)**. *P* < 0.05 in all subgroups.

In the derivation group, we performed sub-group analysis (Table 2, Figure 1). The initial assessment in gender subgroups revealed that women presented higher FMD than men (*p* < 0.05) (Figure 1A), whereas CIMT was not statistically different (*p* > 0.05). In different age groups, we found that younger people had higher FMD (Figure 1B) and lower CIMT (Figure 2A) than older people (*p* < 0.05). Analysis of BMI subgroups revealed that obese people presented lower FMD (Figure 1C) and higher CIMT (Figure 2B) than non-obese individuals (*p* < 0.05). People with hypertension and diabetes showed lower FMD (Figures 1D,E) and higher CIMT (Figures 2C,D) than those without such diseases (*p* < 0.05), while taking drugs or not had no significant effect on FMD or CIMT in population with such diseases (*p* > 0.05). Smokers had lower FMD than non-smokers (*p* < 0.05) (Figure 1F). However, analysis of the population with or without dyslipidemia revealed that there was no significant difference in FMD or CIMT (*p* > 0.05).

**Table 2:** Values of FMD and CIMT in subgroups of gender, age, BMI, hypertension, diabetes, dyslipidemia and smokers.

	**FMD (%)**	***P*-value**	**CIMT (mm)**	***P*-value**
Gender				
Male	6.1 (4.9, 7.0)	0.01	0.6 (0.5, 0.6)	0.21
Female	6.7 (5.7, 7.6)		0.6 (0.5, 0.7)	
Age				
≤ 30	7.0 (5.8, 8.0)	0.00	0.5 (0.5, 0.6)	0.00
>30, ≤ 39	6.4 (5.5, 7.2)		0.6 (0.5, 0.7)	
>40	5.6 (5.1, 6.5)		0.7 (0.6, 0.8)	
BMI				
<24	10.3 (8.5, 11.9)	0.00	0.5 (0.4, 0.6)^a^	0.00
≥24, <28	7.9 (6.7, 8.3)		0.4 (0.4, 0.5)^a^	
≥28	6.1 (5.4, 7.0)		0.6 (0.5, 0.7)^b^	
Hypertension				
Yes	5.7 (5.0, 6.6)	0.00	0.7 (0.5, 0.8)	0.00
Taking drugs	5.6 (4.8, 6.7)	*0.26*	0.8 (0.6, 0.8)	0.02
Not taking drugs	6.0 (5.2, 6.8)		0.6 (0.5, 0.7)	
No	6.8 (5.7, 7.6)		0.6 (0.5, 0.6)	
Diabetes				
Yes	5.7 (5.3, 6.5)	0.00	0.6 (0.6, 0.7)	0.00
Taking drugs	5.6 (5.0, 6.7)	0.96	0.6 (0.5, 0.7)	0.83
Not taking drugs	5.7 (5.3, 6.3)		0.6 (0.6, 0.8)	
No	6.9 (5.8, 7.9)		0.6 (0.5, 0.6)	
Dyslipidemia				
Yes	6.3 (5.6, 7.3)	0.40	0.6 (0.5, 0.7)	0.21
Taking drugs	6.5 (6.5, 7.1)	0.40	0.8 (0.8, –)	0.04
Not taking drugs	6.3 (5.6, 7.3)		0.6 (0.5, 0.7)	
No	6.7 (5.3, 8.0)		0.6 (0.5, 0.7)	
Smokers				0.22
Yes	6.0 (5.2, 6.9)	0.01	0.6 (0.5, 0.7)	
No	6.8 (5.6, 7.9)		0.6 (0.5, 0.7)	

*Data are shown as the median (interquartile range)*;

a *Compared to*

b *BMI ≥ 28, p < 0.05*.

*FMD, flow-mediated dilation; CIMT, carotid intima media thickness; BMI, body mass index*.

### Correlation and Univariate Analysis

Before constructing the prediction equation, we first looked for variables that were clearly related to FMD. We performed bivariate correlation analysis and univariate regression analysis on the above variables and FMD, and screened out 16 potential risk factors (*p* < 0.01) after excluding multicollinearity issues, as shown in Table 3. Multivariate logistic regression was later performed to screen for significant variables associated with FMD.

**Table 3:** The correlation and univariate analysis of FMD and 16 variables.

**Characteristics**	**r**	***P*-value**	**R2**	***P*-value**
BMI	−0.602	0.00	0.362	0.00
WHR	−0.592	0.00	0.350	0.00
γ-GT	−0.462	0.00	0.213	0.00
CP	−0.406	0.00	0.165	0.00
MAP	−0.405	0.00	0.164	0.00
CIMT	−0.374	0.00	0.140	0.00
AST	−0.350	0.00	0.123	0.00
SUA	−0.347	0.00	0.121	0.00
ALT	−0.346	0.00	0.120	0.00
Age	−0.325	0.00	0.106	0.00
HR	−0.318	0.00	0.101	0.00
Insulin	−0.317	0.00	0.101	0.00
LDL	−0.302	0.00	0.092	0.00
FBG	−0.243	0.00	0.059	0.00
smoking	–	–	0.046	0.00
Gender	–	–	0.045	0.01

*BMI, body mass index; WHR, waist-to-hip ratio; γ-GT, γ-glutamyl transpeptidase; CP, C-peptide; MAP, mean arterial pressure; CIMT, carotid intima media thickness; AST, aspartate aminotransferase; SUA, serum uric acid; ALT, alanine aminotransferase; HR, heart rate; LDL, low-density lipoprotein; FBG, fasting blood glucose*.

### Equation Development

After multiple variables combination and modification, we finally selected the following variables for modeling (stepwise, *p* < 0.01), and the expression was:


$$\begin{array}{l} \text{FMD}=\;-0.096\text{BMI}-0.069\text{age}-4.551\text{WHR}\\ \;-0.015\text{AST}-0.242\text{LDL}+17.938 \end{array}$$

**Figure 3:**
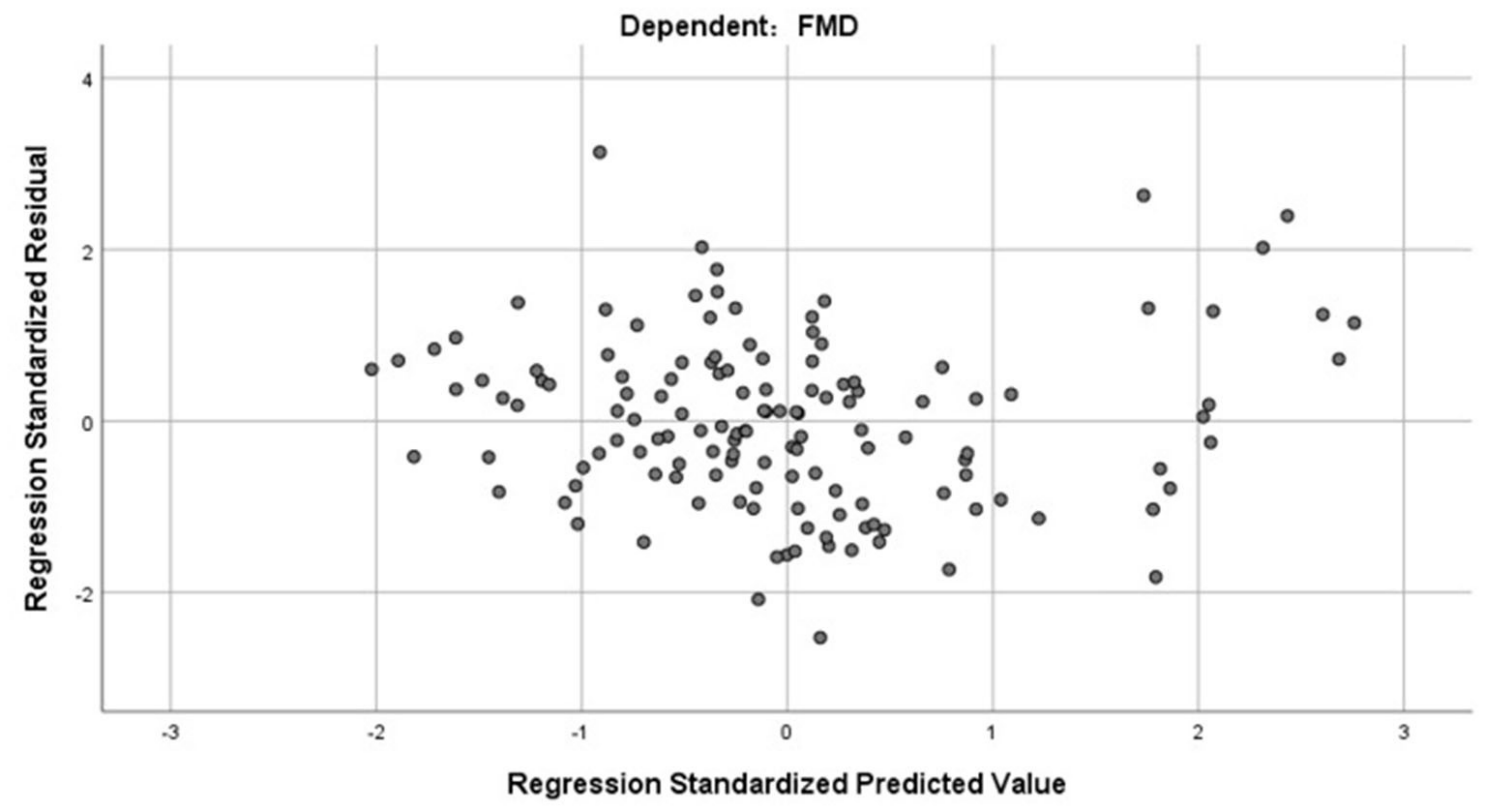
The residual scatter plot of standardized predicted value and standardized residual.

**Figure 4:**
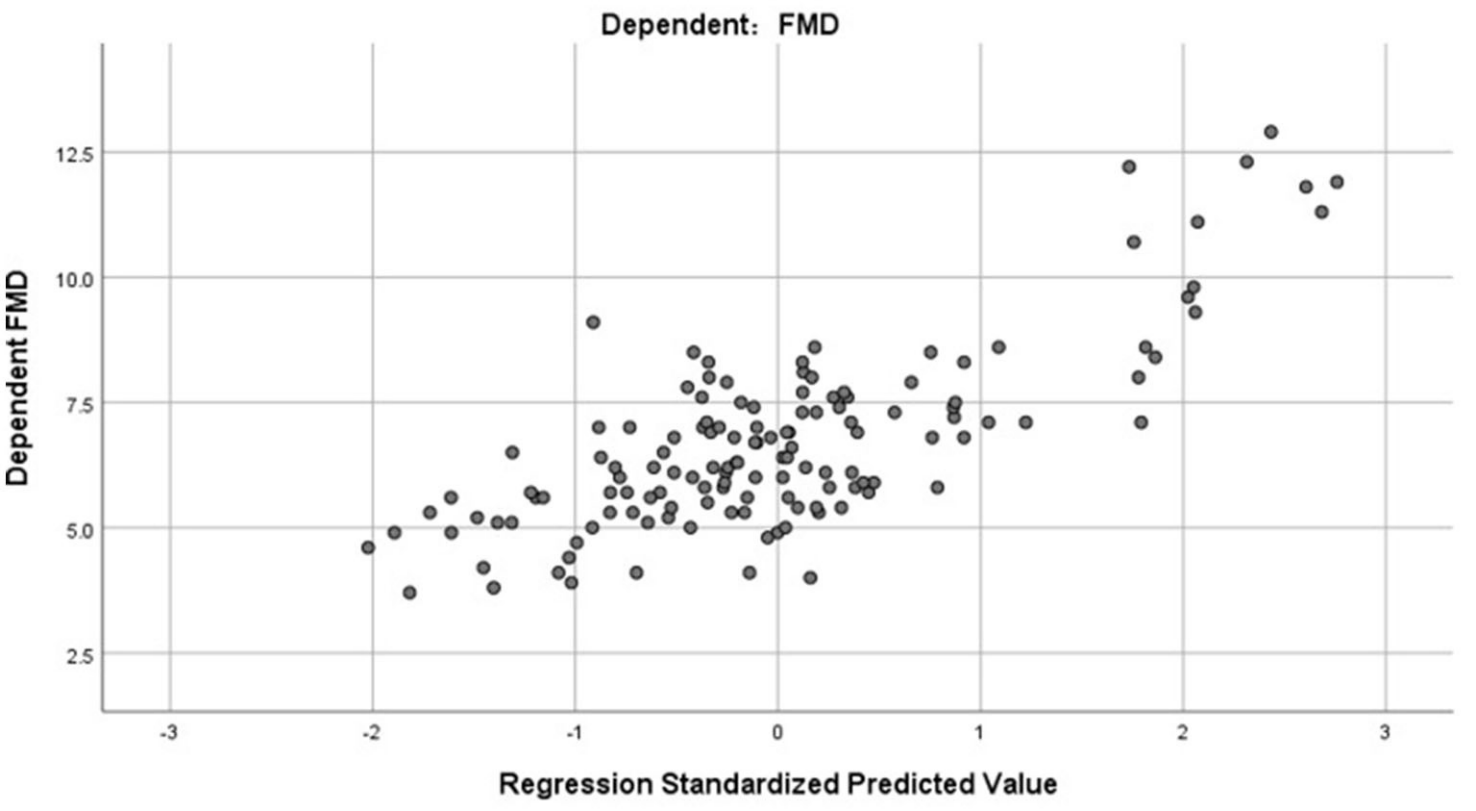
The scatter plot of standardized predicted value and dependent FMD.

R2 = 0.598, and adjusted R2 = 0.582. The regression coefficient and 95% CI of each independent variable are shown in Table 4. The independence test of model residuals, i.e., Durbin-Watson test, was 2.184, which indicates good independence between variables. The collinearity analysis show that the VIF values between the independent variables were <2, which indicates that there was no synteny problem among the four independent variables that we introduced. In addition, we draw a residual scatter plot with the standardized predicted value on the X-axis and the standardized residual on the Y-axis (Figure 3). The scatter points were randomly distributed, and the slope was almost zero. We believe that there was no possibility of autocorrelation. The scatter plot of the standardized predicted value and dependent FMD shows a linear trend (Figure 4).

**Table 4:** The regression coefficient and 95% CI of the equation.

**Variables**	**Coefficients**	**95% CI**	***P*-value**
BMI	−0.096	(−0.124, −0.068)	0.000
Age	−0.069	(−0.095, −0.043)	0.000
WHR	−4.551	(−7.249, −1.853)	0.000
AST	−0.015	(−0.027, −0.003)	0.001
LDL	−0.242	(−0.463, −0.020)	0.003
Constant	17.938	(15.765, 20.111)	0.000

*95% CI, 95% confidence intervals; BMI, body mass index; WHR, waist-to-hip ratio; AST, aspartate aminotransferase; LDL, low-density lipoprotein*.

### Verification of Equations

**Figure 5:**
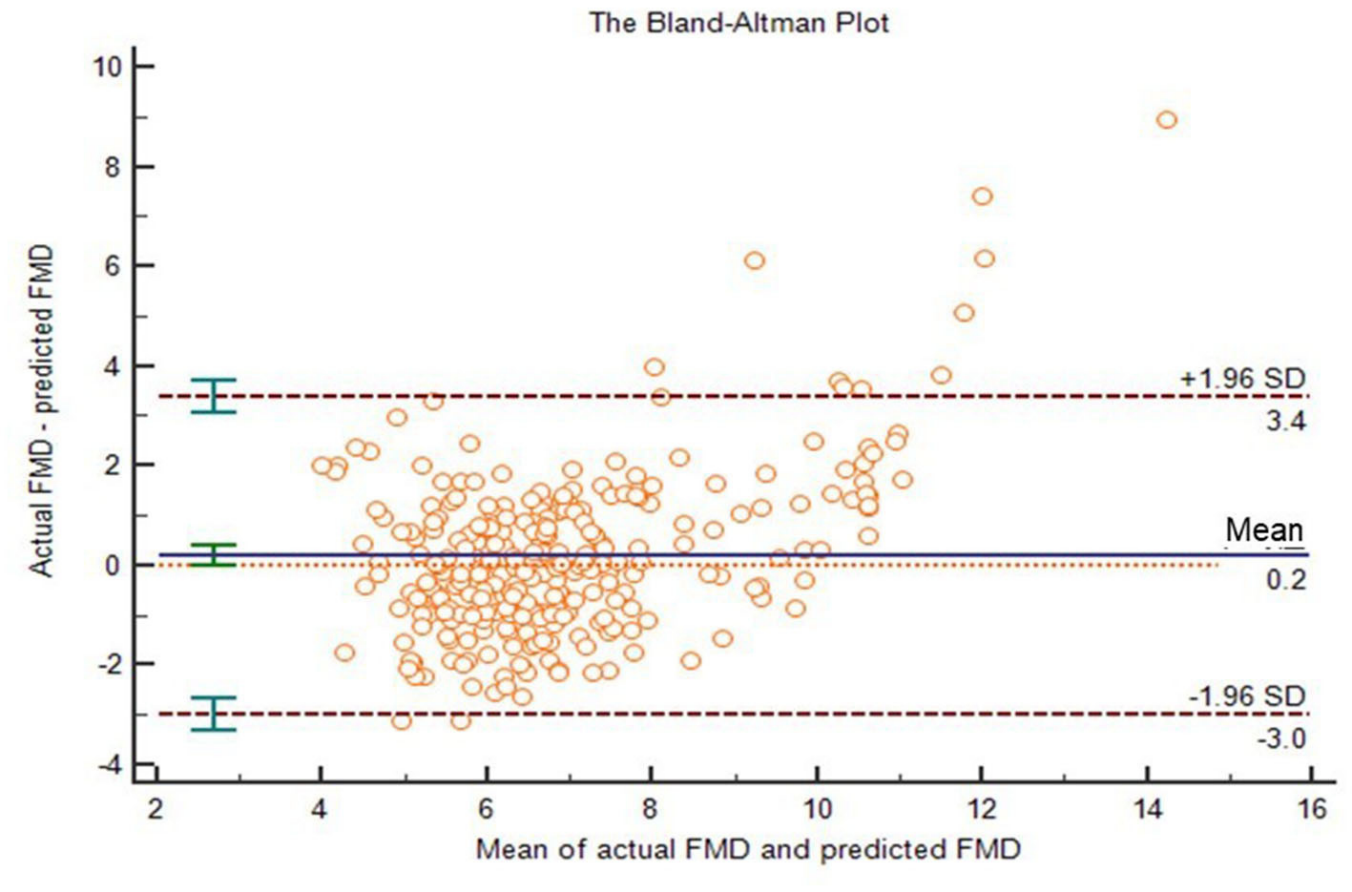
The Bland-Altman plot of actual FMD and predicted FMD. The upper and lower horizontal dotted lines in the picture represented the 95% limits of agreement. The horizontal solid line in the middle represented the average value of the difference. The middle horizontal dotted line indicated the position where the average value of the difference was zero.

To verify the effectiveness of our model, we compared the actual FMD values to predicted FMD values in the verification group. ICC of them was 0.767 (95% CI: 0.704–0.816; *p* < 0.001). The two-related-sample test (Wilcoxon) shows that there was no statistically significant difference between the actual FMD and the predicted FMD (*P* > 0.05). Next, we drew a Bland–Altman plot (Figure 5), which shows that the average value of the differences (middle horizontal solid line) was close to zero (middle horizontal dotted line). Most of the differences between actual FMD and predicted FMD were within the 95% limits of agreement, and only 3% (13/420) of the points lied outside it. We believed that the actual FMD and predicted FMD had high consistency.

**Figure 6:**
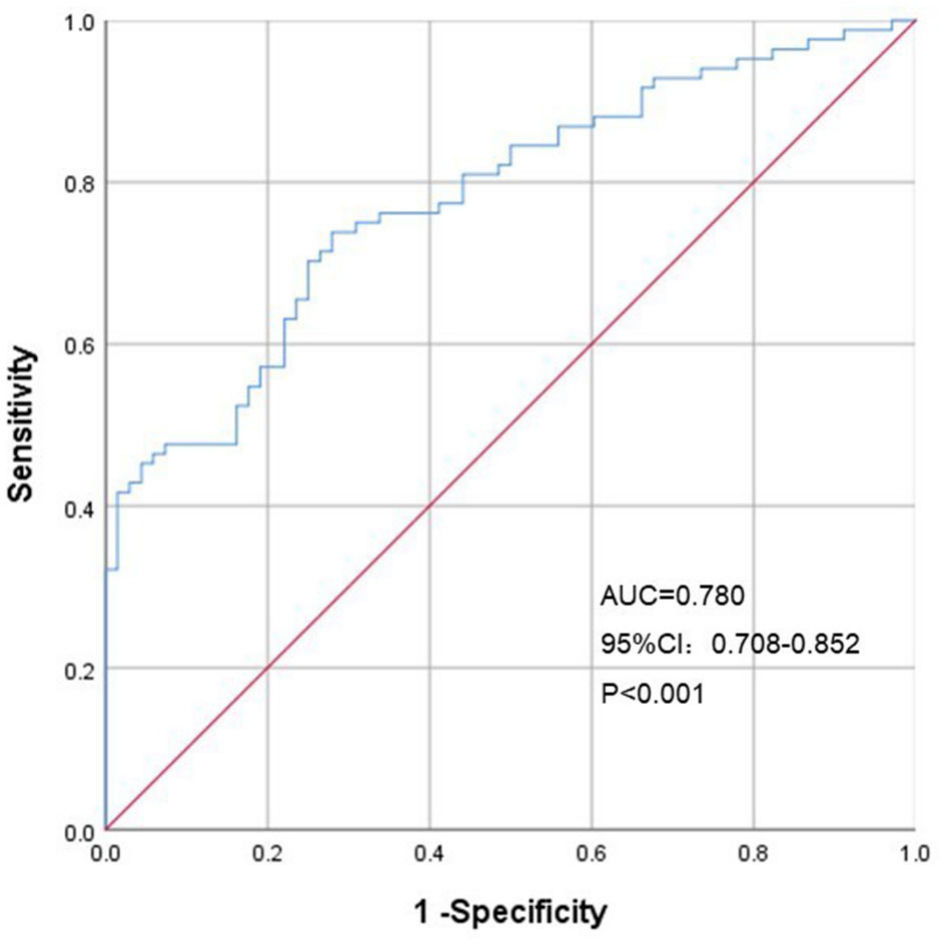
Receiver operating characteristic curves of predicted FMD for Framingham Heart Study CV risk stratification. AUC, area under the curve; CI, confidence interval.

To further evaluate the ability of our prediction model to assess CV risk, we divided the validation cohort into groups according to the Framingham Heart Study CV risk stratification updated in 2008 (10-year CV event risk: 0–6%, 6–20% and > 20%) (10). Our study population had no risk >20%, so it was divided into two groups. The AUC of FMD to identify different CV risk stratification was 0.780 (95% CI: 0.708–0.852, *p* < 0.001), with a sensitivity and specificity of 73.8 and 72.1%, respectively (Figure 6).

## Discussion

The main purpose of this article was to find the influencing factors of vascular endothelial function and establish a simple predictive model of FMD. Endothelial damage is often used as an early marker of various diseases, such as obesity, hypertension, diabetes, and AS. Many studies have shown that FMD can provide important prognostic information beyond traditional CV risk factors (14), and low FMD strongly predicts CV events (15). Therefore, early evaluation of endothelial function is of great significance in diagnosing or monitoring disease progression.

We divided the target population into a derivation group and a verification group through simple random sampling. The ratio of the two was 1:2, which ensured the reliability of the prediction model and reduced the phenomenon of overfitting. The balance test was first performed between them to ensure a random distribution of the indicators. Table 1 shows that their *p* values were both >0.05, which indicates that there was no significant difference in parameters between them.

We first analyzed the relationship between gender and FMD and found that men had lower FMD than women. Study revealed atherosclerotic CVD prevalence in men was greater than in women until menopause, and some studies showed earlier decrements in endothelial function in men compared to women (16, 17). But sex differences in endothelial function was controversial and others showed similar age-related declines between the sexes (18). After menopause, women may have a higher prevalence of CVD compared to age-matched men (19). This may be due to the lack of protective effects of estrogen. There were few postmenopausal women in our participants (total population: 6/420), the results showed that there was no statistical difference in FMD between men and women (*p* > 0.05), but the representativeness was insufficient, and the sample size will be expanded in the future, hoping to conduct in-depth research. The prevalence of CVD increases with age in both men and women. CVD in aging is partly a consequence of the vascular endothelial cell senescence and associated vascular dysfunction (20). Vascular oxidative stress and low-grade inflammation increase with age and is the key mechanism of endothelial diastolic dysfunction (21). Simultaneously, the reduction of estradiol associated with aging (postmenopausal females) (22) and epigenetic modifications (23) may also cause endothelial dilation disorders. This represents a major link between aging and cardiovascular risk.

Many studies have demonstrated impaired endothelial function in obesity, so we compered the FMD values in different BMIs. We found that the group with higher BMI had lower FMD and higher CIMT. This suggested the dual damage of vascular endothelial function and structure related to obesity. Prior study of the impact of obesity revealed FMD decreased in the moderately obese compared with the non-obese (24). This was consistent with our results. An increased CIMT had also been reported in obese patients and weight loss was associated with a reduction in CIMT, consistent with a lowering in risk of cardiovascular events (25, 26). And also, we found that people with diabetes, hypertension and smoking showed lower FMD, all of which suggested an association between FMD and cardiovascular risk factors (12, 27, 28).

Then, we performed bivariate correlation analysis and univariate regression analysis to screen out potential risk factors (*p* < 0.01) to the multivariate logistic regression. After multiple variables combination and modification, we developed a prediction model of FMD, which consisted of BMI, age, WHR, AST and LDL. BMI is a commonly used index to measure the degree of obesity, and BMI≥24 is considered overweight in the Asian population. As we said before, overweight and obesity are factors closely related to vascular endothelial damage (29). Obesity-induced long-term hypoxia, chronic inflammation, oxidative stress and mitochondrial dysfunction are all involved in the development of endothelial dysfunction (30–34). Epigenetics (35) and circulating particles (36, 37) have also been popular mechanisms in recent years. However, obesity-related endothelial damage involves the joint participation of multiple mechanisms, which awaits more in-depth research.

WHR is an important indicator to determine central obesity, and the latter is closely related to an increased risk of CV diseases, even among people with normal BMI (38). Pear-shaped females with normal weight but more fat in the hips and thighs have a lower risk of heart disease and stroke. Fat accumulated in the buttocks and thighs is subcutaneous fat, which has a protective effect. However, the fat of central obesity accumulates in the abdomen, which is visceral fat, and easily releases fatty acids into the blood to cause pathological conditions such as high cholesterol and insulin resistance. This pathological process easily induces early endothelial damage (39, 40).

AST is generally considered a marker of myocardium and liver damage. Perticone et al. reported that AST was closely related to the endothelial function, as evaluated by strain-gauge plethysmography, in both univariate linear analysis and a stepwise multivariate regression model (41). This is consistent with our study. Initial endothelial injury is characterized by endothelial glycocalyx injury, which mediates the release of Syndecan-1 from the endothelium and causes an increase in circulating concentration (42). Researchers found that the concentration of Syndecan-1 was significantly related to AST, and there was an obvious positive correlation between them (43). However, the specific mechanism between AST and vascular endothelial function cannot be fully explained at present, and chronic systemic effects and interorgan communication may promote development.

An increase in pro-atherogenic LDL and its oxidative modifications (ox-LDL) is well known to be a crucial factor in endothelial damage, a key early step and a predictor of the development of AS (44). When the body is continuously exposed to high levels of LDL, inflammatory pathways in vascular endothelial cells are activated to increase local and systemic inflammation, endothelial cell dysfunction and apoptosis, and smooth muscle cell proliferation, resulting in foam cell formation and genesis of AS plaque (45, 46). Therefore, dyslipidemia-induced hyperlipidemic stress is widely recognized as a powerful pathophysiological driver of AS.

However, analysis of these four risk factors also implies that we can improve the function of endotheliocytes through early intervention such as weight loss, a healthy diet and exercise. In some obese people with low FMD, physical exercise or bariatric surgery can increase FMD while losing weight, which suggests the recovery of endothelial injury (14, 47, 48). Aging of endotheliocyte is also not static. Researchers have found that aerobic exercise can significantly prevent endothelial cell aging and change the state of inflammation in the body, especially in elderly individuals (49, 50). The aging rate of endothelial cells after exercise is much lower than that of sedentary peers (51). Mitochondrial-targeted antioxidant supplementation may also play a role in improving age-related vascular dysfunction (52).

Impaired FMD has been associated with conditions predisposing to AS and CVD and represents an early step in the development of subclinical target organ injury and late clinical events (53). Therefore, we intended to evaluate our prediction model in identifying CVD risk stratification. The result showed that FMD model can well identify group with higher CVD risk (AUC = 0.780, p<0.001). Several studies have demonstrated the prognostic value of FMD for CVD events (54, 55). However, some scholars believe that the reproducibility of FMD is low and currently not recommend for the assessment of CV risk. That partly because poor standardization between laboratories and lack of guideline adherence. At present, evidence on these issues pertaining to FMD is incomplete. If FMD can be standardized, it may be an important supplement to CV risk stratification.

The 1:2 grouping method improved the adaptability of our equation. It was easy to operate and very suitable for primary hospitals. On one hand, chronic diseases such as hypertension, diabetes, and obesity occupy the majority of community diseases. Doctors in the community are the “gatekeepers” of the health care system, so monitoring and long-term follow-up of such diseases are very important. On the other hand, basic hospitals are not equipped with sufficient hardware facilities, especially expensive and high-tech machines such as FMD equipment. Thus, a simple and reliable evaluation model as an auxiliary method is of great significance.

Our study had several limitations. First, the sample for model construction and verification was relatively small and data was limited to Chinese individuals. In the future, a prediction model will require a larger sample for verification and we hope to extrapolate the model to other ethnic groups. Second, this was a single-center study. Although the ratio of our training group to the validation group was 1:2 to maximize the generalizability of our model, establishing a standardized and accurate model requires the joint participation of multi-center research. Third, the accuracy of our prediction equation was quite modest. It may be suitable for epidemiological purpose or for primary hospitals. Furthermore, there were differences in the ratio of sex and BMI, and the percentage of males and the non-obese was relatively small in our studied population. In response to these problems, our follow-up research must expand the sample size and maintain the internal balance of each variable.

## Conclusion

Short-term changes or longer-term improvements of vascular endothelial function in interventional trials suggest protective or damaging effects. Therefore, using FMD as a surrogate endpoint to observe changes in endothelial function is timely and simple. We explored the influencing factors of FMD and developed a simple prediction equation. This model will greatly simplify clinical work, but further validation in external populations remains necessary.

## Data Availability Statement

The data analyzed in this study is subject to the following licenses/restrictions: Requests to access these datasets should be directed to 1627486196@qq.com.

## Ethics Statement

The studies involving human participants were reviewed and approved by Chinese Clinical Trial Registry ChiCTR2100041860. The patients/participants provided their written informed consent to participate in this study.

## Author Contributions

XL drafted the manuscript. HL performed the statistical analysis. YZ drafted the figure and legend. YG and LS wrote sections of the manuscript. WB and HY designed the outline of the topic and helped on revising the manuscript. All authors contributed to the article and approved the submitted version.

## Funding

This project was funded by National Key R&D Program (2021YFC2009100), Shanghai Science and Technology Commission (21Y11910900), Shanghai Sixth People's Hospital Surface Cultivation Project (ynms202110), and Shanghai Pujiang Program (2019PJD036).

## Conflict of Interest

The authors declare that the research was conducted in the absence of any commercial or financial relationships that could be construed as a potential conflictof interest.

## Publisher's Note

All claims expressed in this article are solely those of the authors and do not necessarily represent those of their affiliated organizations, or those of the publisher, the editors and the reviewers. Any product that may be evaluated in this article, or claim that may be made by its manufacturer, is not guaranteed or endorsed by the publisher.

## Supplementary Material

Supplementary Figure 1 The scatter plot of the predicted FMD value and the actual FMD value.

Supplementary Figure 2 The scatter plot of age and FMD.

Supplementary Figure 3 FMD in 3 different age groups and different genders.

Supplementary Figure 4 FMD in 4 different age groups and different genders.

Supplementary Figure 5 A figure (example captured in one patient of the study sample) of a FMD acquisition.
