# Supplementary material for: A Prediction Equation to Estimate Vascular Endothelial Function in Different Body Mass Index Populations
Source: Front Cardiovasc Med. 2022 Mar 10;9:766565. doi: 10.3389/fcvm.2022.766565 (PMC8960173; doi:10.3389/fcvm.2022.766565)
Supplement: Supplementary Figure 1 — The scatter plot of the predicted FMD value and the actual FMD value. [file Data_Sheet_1.docx]

Supplementary Material

# Supplementary Figures and Tables

## Supplementary Figures

##
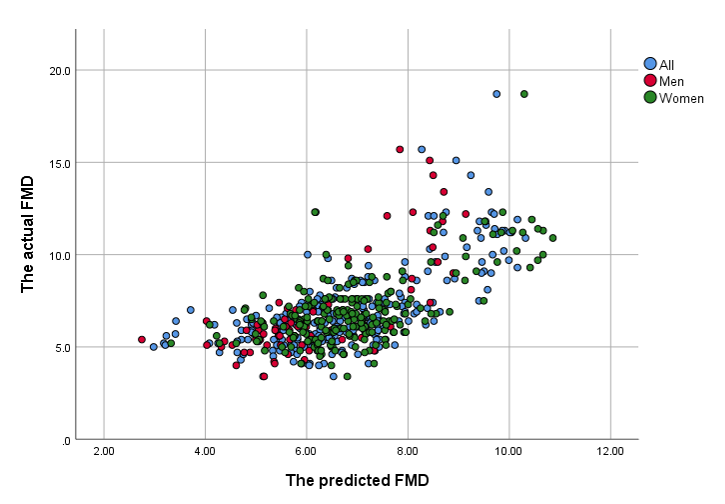


## Supplementary Figure 1. The scatter plot of the predicted FMD value and the actual FMD value in men,

## women and both genders.


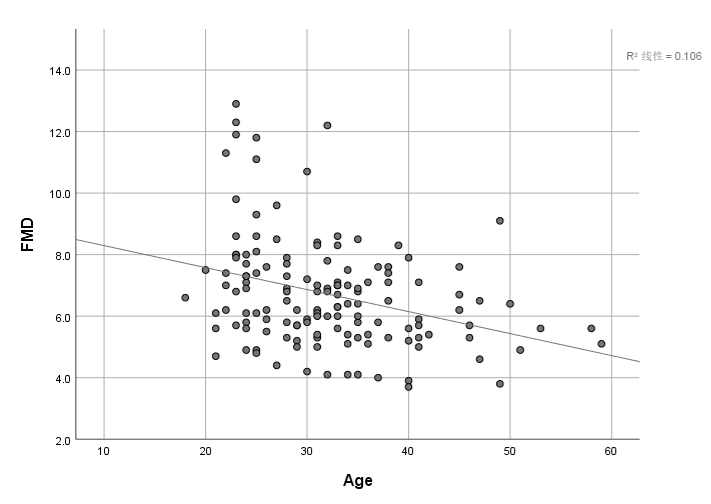


## Supplementary Figure 2. The scatter plot of age and FMD.


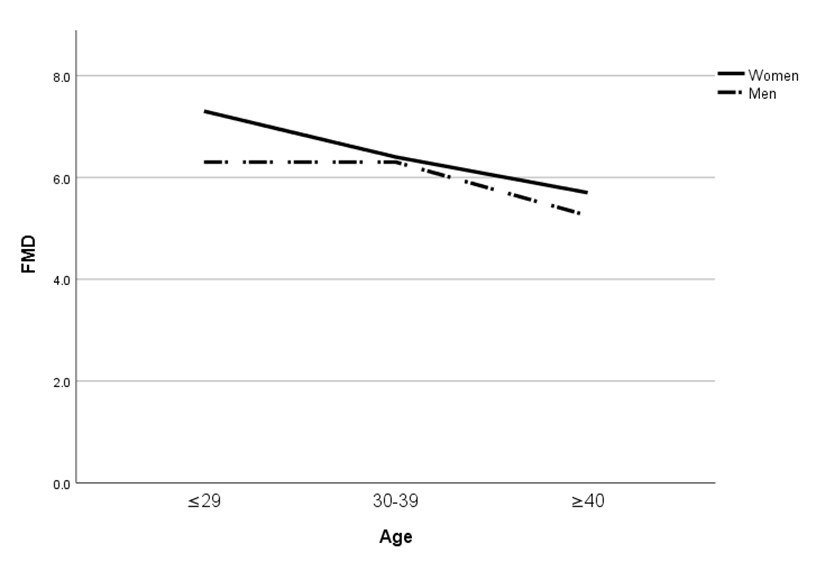


**Supplementary Figure 3**. FMD in 3 different age groups and different genders.


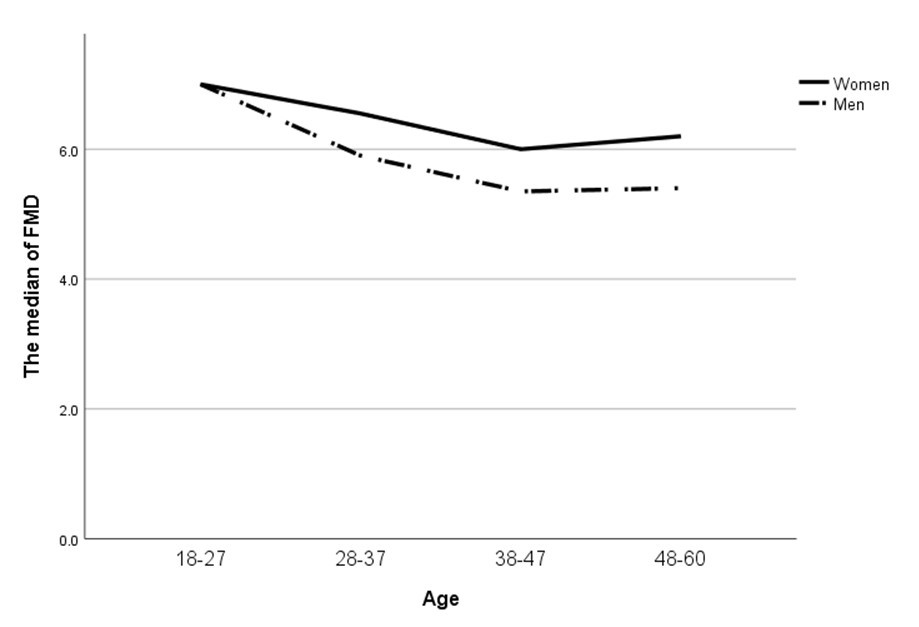


**Supplementary Figure 4**. FMD in 4 different age groups and different genders.


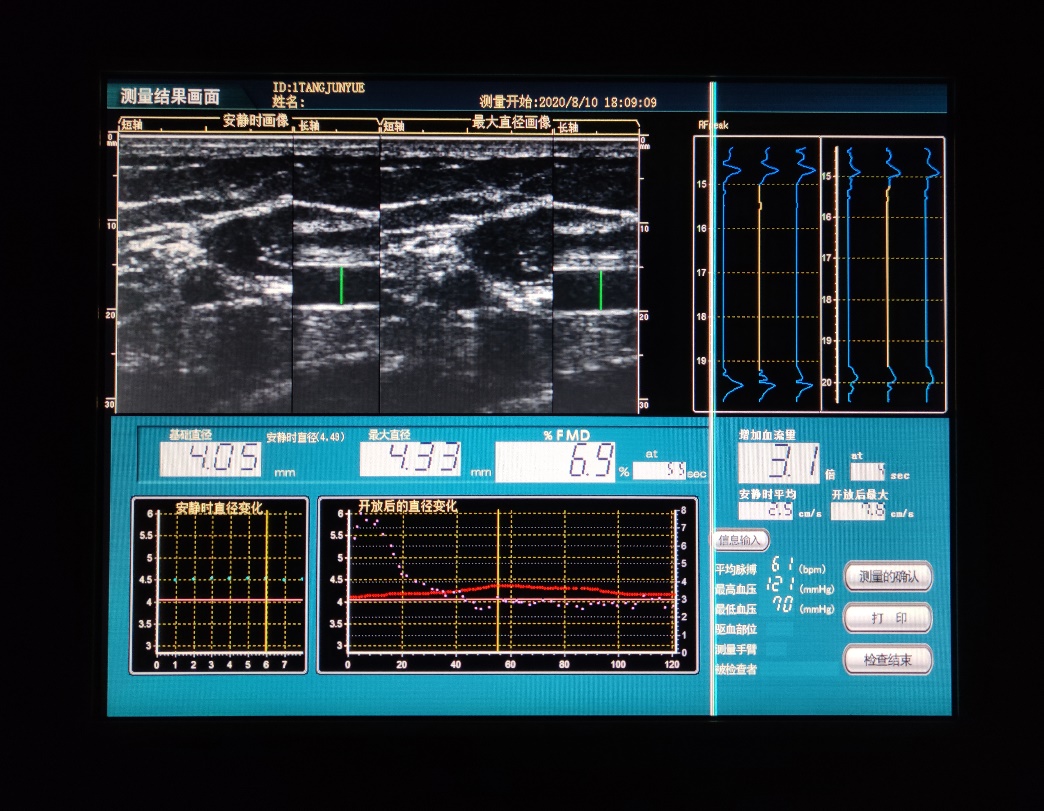


**Supplementary Figure 5**. A figure (example captured in one patient of the study sample) of a FMD acquisition.

## Supplementary Tables

**Supplementary Table 1**. The regression coefficient and 95% CI of the equation for men.

| Variables | Coefficients | 95% CI | P-value |
| --- | --- | --- | --- |
| RFM | -.177 | (-.237, -.118) | .000 |
| FBG | -.175 | (-.317, -.034) | .000 |
| Constant | 13.023 | (10.951, 15.095) | .017 |

95% CI=95% confidence intervals, RFM=Relative Fat Mass, FBG==fasting blood glucose.

RFM= 64 − (20 × height/waist circumference) + (12 × gender)

[male: sex = 0, female: sex = 1]

**Supplementary Table 2** The regression coefficient and 95% CI of the equation for women.

| Variables | Coefficients | 95% CI | P-value |
| --- | --- | --- | --- |
| RFM | -.183 | (-.223, -.143) | .000 |
| Age | -.071 | (-.105, -.037) | .000 |
| ALT | -.010 | (-.016, -.014) | .001 |
| Constant | 17.742 | (15.812, 19.672) | .000 |

95% CI=95% confidence intervals, RFM=Relative Fat Mass, ALT=alanine aminotransferase.

RFM= 64 − (20 × height/waist circumference) + (12 × gender)

[male: sex = 0, female: sex = 1]

**Supplementary Table 3** Characteristics in the normal FMD (≥7.1) and abnormal FMD (< 7.1) cohorts.

| Characteristics | FMD≥7.1 | FMD < 7.1 | P-value |
| --- | --- | --- | --- |
| N (M/F) | 49 (8/41) | 91 (30/61) | 0.04 |
| Age (years) | 29.3 ± 6.8 | 33.5 ± 8.4 | 0.00 |
| BMI (kg/m^2^) | 30.0 ± 7.7 | 38.5 ± 7.0 | 0.00 |
| HR (beats/min) | 82.1 ± 13.2 | 89.5 ± 11.8 | 0.00 |
| SBP (mmHg) | 122.3 ± 16.8 | 135.1 ± 19.7 | 0.00 |
| DBP (mmHg) | 80.8 ± 10.5 | 88.6 ± 13.3 | 0.00 |
| MAP (mmHg) | 94.6 ± 11.5 | 104.1 ± 14.5 | 0.00 |
| WHR | 0.9 (0.8, 1.0) | 1.0 (1.0, 1.1) | 0.00 |
| CIMT (mm) | 0.5 (0.4, 0.6) | 0.6 (0.5, 0.7) | 0.00 |
| TG (mmol/l) | 1.0 (0.6, 1.7) | 1.5 (1.1, 2.5) | 0.00 |
| TC (mmol/l) | 5.0 (4.3, 5.8) | 5.2 (4.5, 6.0) | 0.27 |
| HDL cholesterol (mmol/l) | 1.3 (1.2, 1.5) | 1.2 (1.1, 1.4) | 0.06 |
| LDL cholesterol (mmol/l) | 2.9 ± 0.9 | 3.9 ± 0.9 | 0.00 |
| ALT (U/L) | 23.0 (15.0, 43.0) | 50.5 (30.8, 79.3) | 0.00 |
| AST (U/L) | 18.0 (15.0, 25.0) | 28.5 (20.0, 45.3) | 0.00 |
| γ-GT (U/L) | 19.0 (11.0, 33.0) | 43.0 (29.8, 61.3) | 0.00 |
| ALP (U/L) | 65.0 (52.0, 87.0) | 77.0 (65.5, 93.0) | 0.00 |
| PAB (mg/L) | 265.0 (235.0, 305.0) | 275.5 (242.3, 320.3) | 0.22 |
| Scr (mg/dL) | 64.2 (54.9, 72.0) | 61.6 (51.6, 75.0) | 0.27 |
| SUA (mg/dL) | 344.0 (281.0, 433.0) | 408.5 (345.5, 464.0) | 0.00 |
| BUN (mmol/L) | 4.3 (3.8, 5.2) | 4.8 (4.0, 5.6) | 0.09 |
| RBP (mg/L) | 34.0 (29.0, 42.0) | 39 (32.0, 46.0) | 0.01 |
| CysC (mg/L) | 0.7 (0.6, 0.8) | 0.7 (0.6, 0.8) | 0.21 |
| FBG (mmol/l) | 5.0 (4.6, 5.4) | 5.8 (5.2, 7.5) | 0.00 |
| HbA1c (%) | 5.4 (5.2, 5.7) | 6.0 (5.4, 7.0) | 0.00 |
| Insulin (µIU/ml) | 13.4 (7.6, 31.1) | 25.9 (18.7, 36.8) | 0.00 |
| CP (ng/ml) | 2.8 (1.7, 4.4) | 4.0 (3.2, 4.8) | 0.00 |
| Current smokers (%) | 6 (12.2%) | 26 (28.6%) | 0.03 |
| Hypertension (%) | 6 (12.2%) | 24 (26.4%) | 0.05 |
| Participants on  anti-hypertensives (%) | 4 (8.2%) | 14 (15.4%) | 0.22 |
| Diabetes (%) | 4 (8.2%) | 36 (39.6%) | 0.00 |
| Participants on  anti-diabetics (%) | 3 (6.1%) | 14 (15.4%) | 0.11 |
| Dyslipidemia (%) | 23 (46.9%) | 55 (60.4%) | 0.13 |
| Participants on  lipid-lowering drugs (%) | 1 (2.0%) | 1 (1.1%) | 0.65 |

Normally distributed data were expressed as the mean±standard deviation.

Data not normally distributed were expressed as the median and interquartile range.

Categorical variables were expressed as percentages.

FMD=flow-mediated dilation, M/F=male/female, BMI=body mass index, HR=heart rate, SBP=systolic blood pressure, DBP=diastolic blood pressure, MAP=mean arterial pressure, WHR= waist-to-hip ratio, CIMT=carotid intima media thickness, TG=triglyceride, TC=total cholesterol, HDL=high-density lipoprotein, LDL=low-density lipoprotein, ALT=alanine aminotransferase, AST=aspartate aminotransferase, γ-GT=γ-glutamyl transpeptidase, ALP=alkaline phosphatase, PAB=prealbumin, Scr=serum creatinine, SUA=serum uric acid, BUN=blood urea nitrogen, RBP=retinol-binding protein, CysC=cystatin C, FBG=fasting blood glucose, HbA1c=glycated hemoglobin, CP=C-peptide.

**Supplementary Table 4** The correlation and univariate analysis of FMD and BMI, BRI, AVI, Conl, RFM, ABSI.

| Characteristics | r | P-value | R^2^ | P-value |
| --- | --- | --- | --- | --- |
| BMI | -0.602 | 0.00 | 0.362 | 0.00 |
| BRI | -0.581 | 0.00 | 0.338 | 0.00 |
| AVI | -0.599 | 0.00 | 0.358 | 0.00 |
| ConI | -0.517 | 0.00 | 0.267 | 0.00 |
| RFM | -0.389 | 0.00 | 0.151 | 0.00 |
| ABSI | -0.245 | 0.00 | 0.060 | 0.00 |

FMD=flow-mediated dilation, BMI=body mass index, BRI=body roundness index, AVI=abdominal volume index, ConI=conicity index, RFM=relative fat mass, ABSI=a body shape index.
